# Association of dietary anthocyanidins intake with all-cause mortality and cardiovascular diseases mortality in USA adults: a prospective cohort study

**DOI:** 10.1038/s41598-024-76805-z

**Published:** 2024-11-04

**Authors:** Yifei Yan, Jianchang Li

**Affiliations:** Department of Urology, Affiliated Hospital of Guangdong Medical University, Zhanjiang, 524001 China

**Keywords:** Anthocyanidins, Mortality, Dietary intake, NHANES, Cardiovascular diseases mortality, Nutrition, Public health, Epidemiology, Plant sciences

## Abstract

Anthocyanins, as a type of polyphenolic compound, have been discovered to offer multiple health benefits to humans, including anti-aging and anti-inflammatory properties. This prospective cohort study aims to examine the relationship of dietary intake of anthocyanin with all-cause mortality and cardiovascular diseases mortality in the US. The aim of this research was to explore the possible correlation between anthocyanin consumption and the mortality rate from all causes as well as from cardiovascular disease. Based on the Public Access NHANES-linked National Death Index files up to December 31, 2019, we identified mortality status and heart disease-specific causes of death. A multivariate Cox regression analysis was employed to evaluate the effects of anthocyanin intake on mortality outcomes, generating hazard ratios and 95% confidence intervals, adjusting for various demographic characteristics, lifestyle factors, and comorbid conditions. Additionally, we utilized Kaplan-Meier survival curves, subgroup analyses. In different scenarios, dietary anthocyanin intake was assessed using restricted cubic spline models. A total of 11,959 participants completed the final cohort, averaging 47.12 years of age (SD ± 0.35). Following adjustments for multiple variables, an inverse relationship was identified between anthocyanin intake in the highest quartile and all causes mortality, yielding a hazard ratio (HR) of 0.68 (95% CI: 0.52–0.89). Similarly, elevated anthocyanin consumption was linked to a reduction in heart disease mortality, with HR of 0.61 (95% CI: 0.38–0.97). Additionally, dose–response curve revealed a consistent decrease in both all-cause and cardiovascular mortality with increasing anthocyanin intake. Further subgroup analyses revealed that elevated intake of anthocyanins was linked to decreased all-cause mortality in White individuals and males. Moreover, high anthocyanin intake was significantly correlated with reduced all-cause mortality irrespective of hypertension or hyperlipidemia status. Our research indicates that an appropriate dietary intake of anthocyanins is associated with a reduction in overall mortality rates. Furthermore, the findings reveal a substantial association between anthocyanin intake and decreased mortality from cardiovascular diseases, suggesting that anthocyanins may effectively lower the risk of cardiovascular-related deaths.

**Supplementary Information:**

The online version contains supplementary material available at 10.1038/s41598-024-76805-z.

## Introduction

As a category of polyphenolic substances extensively distributed in berries and pigmented flora^1,2^, anthocyanins have garnered long-standing acknowledgment for their abilities in counteracting aging^3^, inflammation^4^, cancer^5,6^, and safeguarding neural tissues^7,8^. Amidst the prevalent accessibility of calorie-rich foods and the escalating consumption of processed foodstuffs, the weight of diet-related ailments like diabetes, hypertension, and cardiovascular disorders is amplifying^9–11^. Anthocyanins, as non-essential elements inherent in fresh edibles, hold potential in diminishing the pervasiveness of chronic illnesses.

In the realm of anthocyanin classification, six principal subtypes—cyanidin, delphinidin, pelargonidin, petunidin, malvidin, and peonidin—are recognized as representative of the majority of anthocyanins found in plants. Numerous epidemiological investigations and clinical experiments have indicated that these anthocyanins and their subtypes possess significant efficacy in preventing malignant tumors^12,13^, diabetes mellitus^14,15^, cardiovascular disease^16,17^, and other critical illnesses. Fresh foods abundant in anthocyanins, such as blueberries^18^, mulberries^19^, and cherries^20^, have been shown to confer diverse health benefits across multiple human physiological systems.

Although the consensus acknowledges the positive impact of anthocyanins on cardiac function and cardiovascular health, primarily through the stimulation of nitric oxide (NO) release or the neutralization of reactive oxygen species (ROS), there remains a lack of prospective studies on the long-term health effect pertaining to quantitative anthocyanin intake. Prior investigations have put the spotlight on the outcomes of specific plant-based foods in mitigating cardiovascular disease risks or influencing biomarkers, thereby neglecting the comprehensive evaluation of total anthocyanin consumption and its broader health implications, which has created a significant research gap in this domain. Furthermore, some studies report that anthocyanin intake may not exert a pronounced effect on cardiovascular health^21,22^, introducing a considerable degree of uncertainty regarding the relationship between anthocyanins and cardiovascular diseases.

Consequently, the comprehensive wellness benefits in body system of dietary anthocyanins and their protective effects on cardiovascular health remain inconclusive. This study synthesizes data from the National Health and Nutrition Examination Survey (NHANES) for the periods 2007–2010 and 2017–2018 to examine the association between anthocyanin intake and both all-cause mortality and cardiovascular disease-specific mortality. Moreover, we analyzed the dose–response relationship to further elucidate how varying levels of anthocyanin consumption impact these health outcomes.

## Method

### Study design and participants

**Fig. 1:**
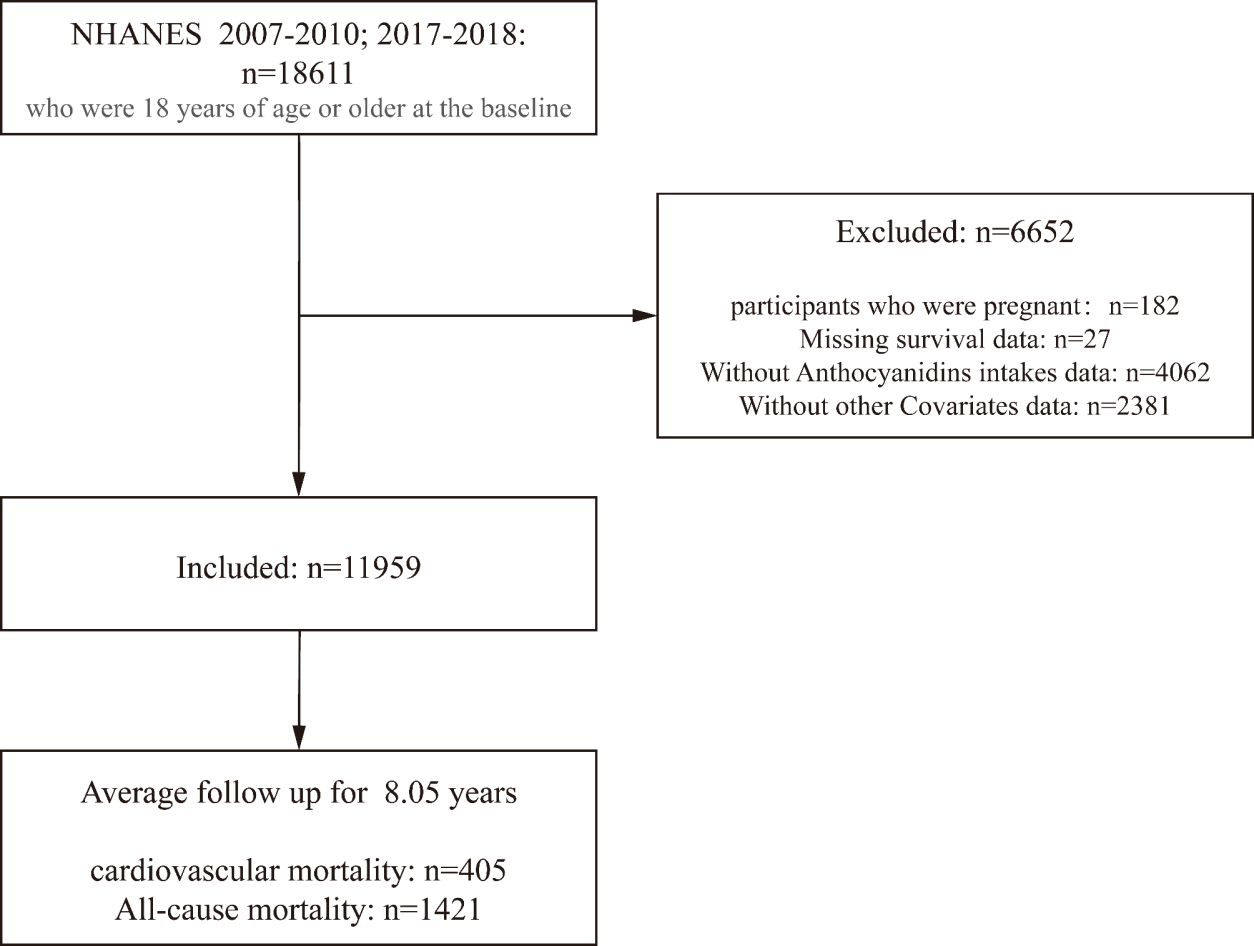
Flowchart of the participants included in the final analysis.

NHANES is a multicenter program to give researcher instant access to information on nationally representative survey based on USA adults. The program was meticulously formulated to appraise the state of health and nutritional situation of the U.S. population systematically. It utilizes a sophisticated, layered, multi-step sampling approach and has been widely used as a sizable long-term study. This sample accurately mirrors the nation demographic composition through connections to a subsequent survey^23^. NHANES data acquisition methodology includes initial in-home interviews, health screenings at mobile examination centers, and the further investigation of telephone interviews. To evaluate the dietary habits of the U.S. population, the intake levels of dietary Anthocyanidins were determined using the Food and Nutrient Database for Dietary Studies developed by USDA. This resource is based on data from the NHANES outcomes. The study received ethical approval from the NHANES review board at the Centers for Disease Control and Prevention, and written informed consent agreements were secured from all participants. A total of 18,611 participants, who were at least 18 years old at the time of the initial assessment, were enrolled in the study during the 2007–2010 and 2017–2018 NHANES cycles. Initially, we excluded 182 participants who were pregnant. Subsequently, we eliminated 27 participants without mortality data. Additionally, we excluded 4,062 participants who did not participate in the dietary questionnaire survey assessing Anthocyanidins intake and 2,381 participants were excluded owing to the deficiency of essential covariate data, which included education, smoking, hypertension, body mass index (BMI), hyperlipidemia, alcohol drinking status, eating healthy index, diabetes mellitus (DM), total energy intake, protein intake, etc. Ultimately, 11,959 participants with complete covariates were selected for inclusion (Fig. 1).

### Dietary anthocyanidin intake

Anthocyanidin intake in this study was based on the consumption of food and beverages, excluding Anthocyanidin medications or supplements. The collected foods were coded using the U.S. Department of Agriculture FNDDS database to calculate primary flavonoid compound intake types for each participant on both the initial and subsequent recording dates. To investigate the relationship between dietary Anthocyanidin intake and human health, this research utilized the average intake of Anthocyanidins, obtained through two 24-hour dietary recalls. The initial dietary data were gathered by experienced interviewers, and the subsequent information was obtained through telephone interviews conducted 3 to 10 days later.

Dietary Anthocyanidin intake was defined as the cumulative sum of six Anthocyanin subclasses intakes: delphinidin, peonidin, malvidin, petunidin, cyanidin and pelargonidin^24^. Participants were categorized into four groups (Q1, Q2, Q3, Q4) based on the quartiles of each Anthocyanidin intake.

### Ascertainment of mortality

A unique study identifier was utilized to ascertain all-cause and cardiovascular disease mortality. This identifier was matched against the National Death Index using a probability matching algorithm developed by the National Center for Health Statistics to determine the survival status of the follow-up population. In cases where no match was found, it was presumed that the individual remained alive as of that date. The follow-up period, calculated in person-years, spanned from baseline to either the participant’s death or the end of the follow-up period on December 31, 2019.

Following the guidelines of the ICD-10, deaths due to all causes and cardiovascular diseases were identified. All-cause mortality encompassed deaths resulting from cardiovascular diseases (codes I00-I09, I11, I13, I20-I51, and I60-I69), malignant neoplasms (codes C00-C97), pneumonia and influenza (codes J09-J18), along with all other causes. Additionally, only participants who died from heart-related causes were included in a separate subgroup analysis to determine whether the independent variable had a distinct effect on the cause of heart-related deaths. For a comprehensive understanding of mortality outcomes, further data is available at the following link: Available from: [https://www.cdc.gov/nchs/data-linkage/mortality-methods.htm]

### Covariate assessment

Our covariates included age, sex, healthy eating index (HEI)^25,26^, ethnicity, education level, BMI, Hyperlipidemia^27^, total energy, protein, carbohydrate and total sugars intake. Smoking status was recorded as never smoked, former smoker, or current smoker (now). Specifically, smoking status was defined as: now (smoked over 100 cigarettes in their lifetime and continues to smoke at present), former (smoked over 100 cigarettes in their lifetime but has quit smoking now), and never (smoked below 100 cigarettes in their lifetime). We have categorized physical activity levels into quartiles based on the Metabolic Equivalent of Task (MET) calculated on a weekly basis. These quartiles are defined as: very low, low, moderate, and high^28^. Alcohol consumption status was categorized as heavy, moderate, mild, former, and never^29,30^.

Diagnosis criterion of Hypertension was identified as self-reported diagnosis, systolic blood pressure (SBP) ≥ 140 mmHg, diastolic blood pressure (DBP) ≥  90 mmHg^31^, or use of antihypertensive drugs. Diabetes Mellitus (DM) and related abnormalities were classified as follows^32,33^: (1) DM: diabetes mellitus; (2) IFG: impaired fasting glycemia; (3) IGT: impaired glucose tolerance; (4) no diabetes. Detailed definitions of general population characteristics, lifestyle habits, and disease conditions are provided in Supplementary Methods.

### Statistical analysis

As a summary of baseline characteristics, continuous variables were presented as averages (standard deviations), while categorical variables were represented as frequencies (percentiles). Quartiles of dietary Anthocyanidin intake were documented (Q1, Q2, Q3, Q4). Follow-up person-years were calculated from the baseline date until the occurrence of death, loss to follow-up, or the study end date (December 31, 2019), whichever occurred first. In all analyses, we applied the specialized dietary weights “wtdr2d” provided by NHANES to ensure accurate data weighting.

We stratified the average anthocyanin intake by age (12 groups: 20–25, 25–30, 30–35, 35–40, 40–45, 45–50, 50–55, 55–60, 60–65, 65–70, 70–75, 75–80 years), BMI (5 groups: <18.5, 18.5–25, 25–30, 30–35, ≥ 35 kg/m²), education level, and ethnicity. To illustrate variations in anthocyanin consumption across different demographic groups, we generated bar charts and spline curves. These visualizations enabled us to analyze trends in average anthocyanin intake among groups with different attributes. Differences in characteristics, disease status, and other parameters between anthocyanin intake quartiles were examined using weighted Pearson’s Chi-squared tests were performed on categorical variables, and the weighted Wilcoxon rank-sum tests were performed on continuous variables.

The relationships between anthocyanin consumption and the risks of all-cause mortality or cardiovascular disease (CVD) mortality were evaluated using a multivariate Cox regression model. This model estimated hazard ratios (HRs) and their 95% confidence intervals (CIs). Three progressively adjusted models were employed: Model 1: Adjusted solely for baseline age (years, continuous). Model 2: Adjusted for ethnicity, age, sex, protein intake, smoking status, BMI (kg/m², continuous), physical activity level, total energy intake, carbohydrate intake, and total sugar intake. Model 3: Adjusted for all covariate, in addition to other mortality risk factors such as DM, hypertension, alcohol consumption, and hyperlipidemia. Additionally, multivariate Cox regression analyses were stratified by sex, education level, BMI group, smoking status, hyperlipidemia, Diabetes Mellitus, and ethnicity. The survey-weighted Likelihood Ratio test was utilized to evaluate the significance of interaction effects.

To visualize the relationship between dietary anthocyanin intake and fatality risk over the follow-up period, weighted Kaplan-Meier survival curves were constructed, stratified by levels of anthocyanin intake. Age-standardized death rates were computed utilizing the population based on the 2000 census as a reference. The relationship between anthocyanin intake and mortality from all causes and cardiovascular disease was also investigated using restricted cubic spline curves (RCS). All statistical analyses were conducted utilizing the “nhanesR” package within R software in version 4.3.2, deemed statistically significant if the P-value is below 0.05.

## Results

### Baseline characteristics

**Fig. 2:**
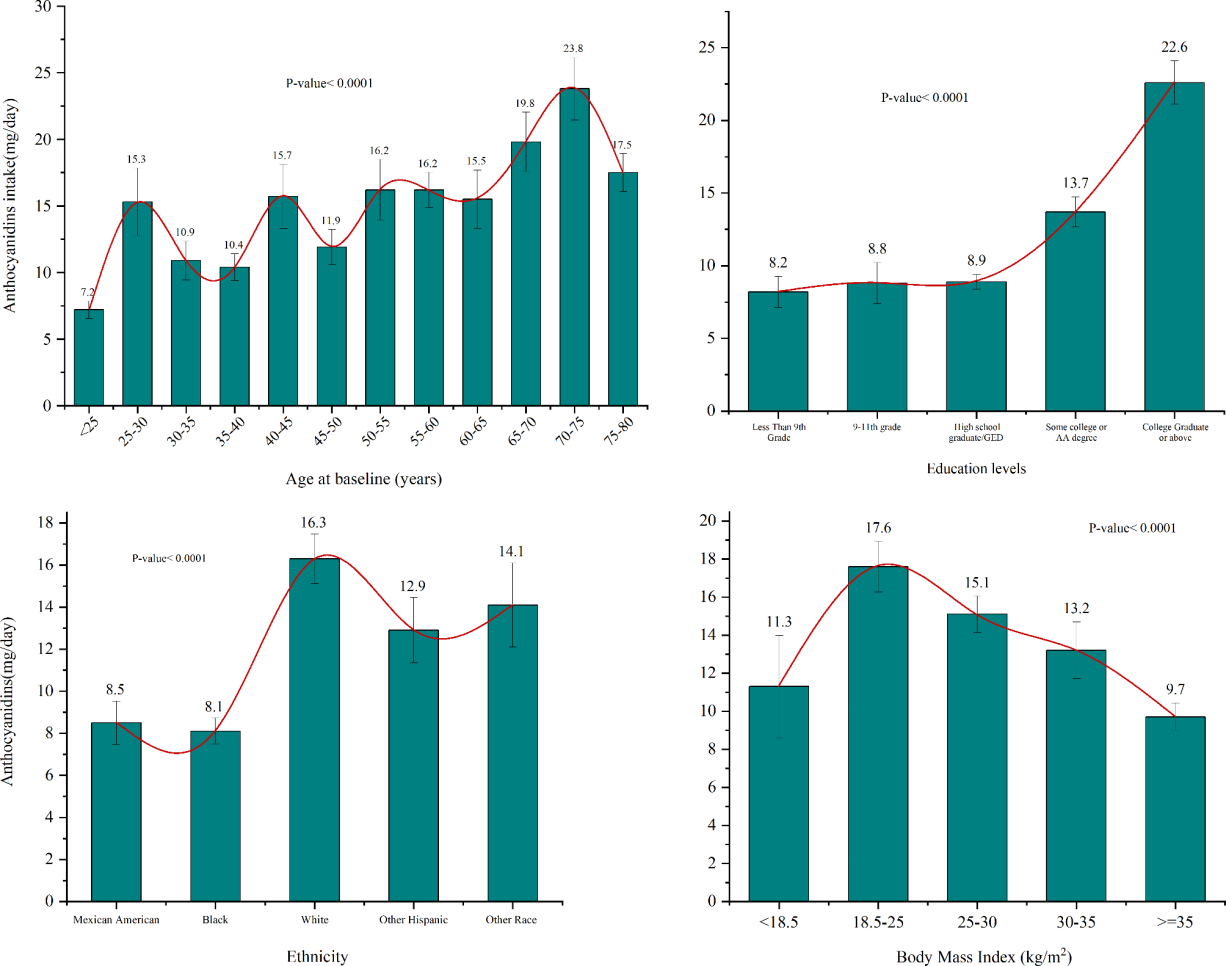
The anthocyanidin intake by baseline characteristics.

According to the established exclusion standards, an aggregate of 11,959 participants were included in the study, with a mean (SD) age of 47.12 (± 0.35) years; of these, 5,814 (48.2%) were female. Over an average follow-up duration of 8.05 years (96,279 total person-years) the study documented 1,421 deaths. Table 1 presents the sample sizes and weighted percentages, along with significance levels for differences within various variable categories, representing 196,230,521 non-hospitalized inhabitants of the United States. Notably, significant differences were observed in demographic characteristics and baseline clinical features between participants with lower Anthocyanidin intake (Q1) and those with higher Anthocyanidin intake (Q4). Participants with higher Anthocyanidin intake tended to have higher total energy intake (*P* = 0.01), higher healthy eating index scores (*P* < 0.0001), and higher protein intake (*P* = 0.01). Survival status was not observed to be significantly associated with higher Anthocyanidin intake (*P* = 0.21). The Anthocyanidin intake was higher in participants who were at a higher level of education, BMI 18.5–25 kg/m^2^, White race, higher age (Fig. 2). Additionally, survival status was used as a baseline characteristic for stratification, as detailed in Supplementary Table 1.

**Table 1:** Characteristics study subjects classified by quartiles of anthocyanidin consumption.

Characteristic^a^	Quintiles of dietary anthocyanidins intake^d^				*P*-value^b^
	Q1	Q2	Q3	Q4	
Number (no.)	3005	2979	2985	2990	
BMI^c^ (kg/m^2^)	30.06 ± 0.20	29.37 ± 0.22	29.25 ± 0.21	28.08 ± 0.16	< 0.0001
HEI-score^c^	42.61 ± 0.39	49.01 ± 0.35	52.97 ± 0.40	57.71 ± 0.38	< 0.0001
Age (years)	43.33 ± 0.41	45.81 ± 0.45	48.18 ± 0.63	50.66 ± 0.60	< 0.0001
Total energy(kcal/day)	2033.43 ± 23.92	2078.68 ± 23.41	2125.97 ± 28.05	2155.02 ± 23.81	0.01
Protein (g/day)	79.57 ± 1.03	81.98 ± 1.04	83.03 ± 1.15	84.52 ± 1.13	0.01
Carbohydrate (g/day)	237.01 ± 3.12	244.58 ± 2.40	258.08 ± 2.95	258.30 ± 2.83	< 0.0001
Total sugars (g/day)	105.05 ± 1.80	106.67 ± 1.70	111.81 ± 1.76	116.65 ± 1.99	< 0.001
*Alcohol status*					< 0.0001
Former	481(12.32)	501(13.64)	474(11.20)	319(8.23)	
Heavy	787(28.69)	635(23.49)	534(19.36)	460(16.69)	
Mild	877(31.73)	966(35.82)	1090(40.97)	1329(45.30)	
Moderate	499(18.62)	453(16.04)	429(16.06)	535(20.79)	
Never	361(8.64)	424(11.02)	460(12.41)	347(8.99)	
*Ethnicity*					< 0.0001
Mexican American	325(6.51)	626(11.62)	608(10.59)	323(5.21)	
Black	794(14.95)	609(11.81)	558(11.52)	454(7.15)	
White	1432(67.51)	1224(63.41)	1250(64.02)	1656(76.29)	
Hispanic	214(3.83)	329(6.61)	371(6.95)	296(4.94)	
Other race	240(7.20)	191(6.55)	198(6.92)	261(6.41)	
*Education*					< 0.0001
9–11th grade	563(13.40)	478(12.19)	416(10.32)	250(5.75)	
College graduate	434(18.87)	504(22.79)	686(30.17)	1050(44.50)	
High school graduate/GED	848(30.43)	758(28.86)	641(23.13)	597(17.90)	
Less than 9th	262(4.50)	388(6.13)	386(6.00)	173(2.90)	
Some college or AA degree	898(32.81)	851(30.03)	856(30.38)	920(28.96)	
*Smoke*					< 0.0001
Former	642(21.37)	741(24.01)	766(24.72)	857(27.47)	
Never	1397(47.81)	1572(54.65)	1748(59.35)	1794(62.46)	
Now	966(30.83)	666(21.35)	471(15.92)	339(10.07)	
*Physical activity level*					< 0.001
Very low	851(22.49)	856(23.09)	783(19.90)	679(18.01)	
Low	702(24.18)	718(24.41)	785(26.16)	833(26.61)	
Intermediate	638(23.34)	654(24.86)	711(27.59)	819(31.43)	
High	814(30.00)	751(27.64)	706(26.36)	659(23.96)	
*Hypertension*					0.51
No	1775(65.41)	1714(64.69)	1728(62.86)	1736(65.39)	
Yes	1230(34.55)	1265(35.31)	1257(37.14)	1254(34.61)	
*Diabetes mellitus*					0.20
DM^c^	533(13.69)	597(13.57)	629(15.52)	493(12.02)	
IFG^c^	160(4.46)	164(5.26)	138(4.07)	143(4.90)	
IGT^c^	78(2.15)	104(2.73)	108(3.19)	113(3.30)	
No	2234(79.70)	2114(78.44)	2110(77.23)	2241(79.78)	
*Hyperlipidemia*					0.58
No	898(31.21)	832(29.15)	817(30.40)	847(31.90)	
Yes	2107(68.79)	2147(70.85)	2168(69.60)	2143(68.10)	
*Status*					0.21
Alive	2631(91.74)	2617(91.97)	2618(91.93)	2672(93.27)	
Death	374(8.26)	362(8.03)	367(8.07)	318(6.73)	

^a^Values are numbers (%) for categorical variables and means ± SDs for continuous variables.

^b^*P*-value for the comparisons between quintiles.

^c^BMI, Body Mass Index; HEI-score, Healthy eating index; DM, Diabetes mellitus; IFG, Impaired Fasting Glycaemia; IGT, Impaired Glucose Tolerance.

^d^The NHANES participants was weighted differently to adjust for the probability of cluster sampling and oversampling of Hispanics and African Americans or aged 60 and above. Therefore the actual number does not match the weighted percentage.

### Anthocyanidin intake and mortality

Utilizing a multivariable adjusted model, it was established that the cohort with the highest anthocyanidin intake (Q4) displayed a reduced total mortality rate in comparison to those with minimal anthocyanidin consumption (Q1). Specifically, the age-adjusted hazard ratio (HR) for all-cause mortality in the highest quartile(Q4) of anthocyanidin intake was 0.48 (95% CI: 0.39–0.59). When further adjustments were made for demographic characteristics and nutrient intake, the HR for all-cause mortality was ascertained to be 0.66 (95% CI: 0.51–0.84) in individuals with high anthocyanidin consumption. Additional adjustments for diseases, nutrient intake, alcohol use, and the healthy eating index revealed a similarly substantial difference, with an HR of 0.68 (95% CI: 0.52–0.89). An analogous correlation was noted regarding cardiovascular disease mortality, with multivariable adjustments yielding an HR of 0.61 (95% CI: 0.38–0.97) for the highest quartile versus the lowest quartile of anthocyanidin intake (*P* = 0.022) (Table 2).

**Table 2:** Hazard ratios (95% CIs) of mortality with weighted anthocyanin.

Characteristic	Quintiles of dietary anthocyanidins intake				*P*-Trend
	Q1	Q2	Q3	Q4	
Participants, no.	3005	2979	2985	2990	NA
Followtime, years	7.52 ± 0.23	7.98 ± 0.17	7.89 ± 0.19	7.46 ± 0.26	
Anthocyanidins intake, median (range), mg/d	0.000 [0.000,0.135]	0.900 (0.135,2.1]	4.735 (2.1,11.412]	27.990 (11.412,756.1]	NA
*All-cause mortality*					
Death/person-years	374/2,176	362/2,139	367/2,209	318/1,958	
Model 1 [HR (95% CI)] ^a^	Referent	0.73(0.60,0.90)	0.61(0.50,0.75)	0.48(0.39,0.59)	< 0.0001
Model 2 [HR (95% CI)] ^b^	Referent	0.83(0.69,1.01)	0.76(0.62,0.94)	0.66(0.51,0.84)	< 0.001
Model 3 [HR (95% CI)] ^c^	Referent	0.83(0.68,1.01)	0.75(0.61,0.93)	0.68(0.52,0.89)	0.004
*Cardiovascular mortality*					
Death/person-years	104/588	95/560	106/648	100/625	
Model 1 [HR (95% CI)] ^a^	Referent	0.75(0.48,1.19)	0.57(0.38,0.85)	0.44(0.30,0.65)	< 0.0001
Model 2 [HR (95% CI)] ^b^	Referent	0.87(0.56,1.35)	0.76(0.51,1.13)	0.64(0.44,0.95)	0.017
Model 3 [HR (95% CI)] ^c^	Referent	0.84(0.52,1.34)	0.72(0.48,1.09)	0.61(0.38,0.97)	0.022

^a^Cox proportional hazard model adjusted for age.

^b^Cox proportional hazards model adjusted by age sex, ethnicity, smoke, BMI, education level, Physical activity level, total energy intake, protein intake, carbohydrate intake, sugars intake.

^c^Further adjusted for alcohol user, healthy eating index, Hypertension, Diabetes mellitus, Hyperlipidemia.

**Fig. 3:**
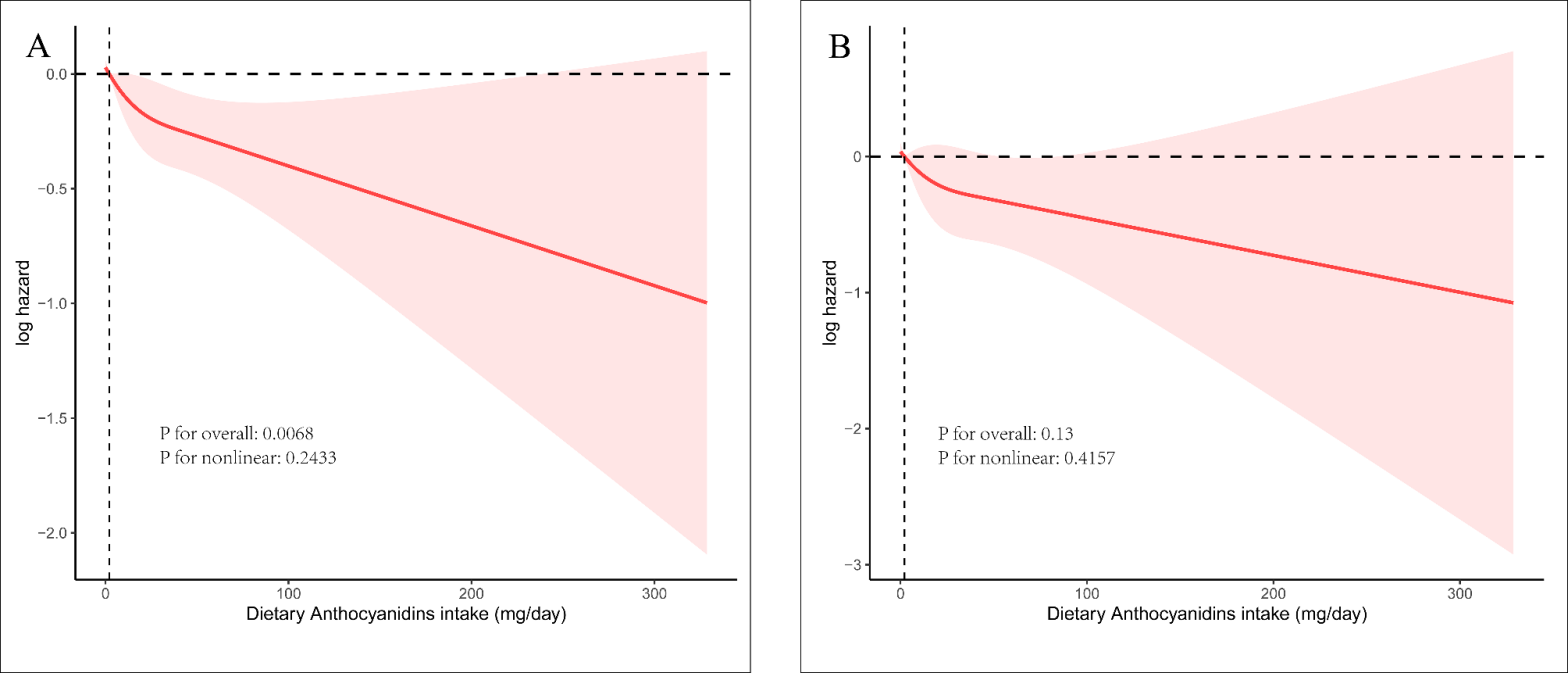
Dose–response association between dietary anthocyanidins intake and risk of mortality. **A** For all-cause mortality, **B** for cardiovascular diseases mortality.

Multivariable adjustment Cox regression incorporating RCS was employed to investigate the dose–response relationship between dietary anthocyanin consumption and the risk of mortality. The analysis revealed a monotonic decreasing trend in both all-cause mortality (P for overall = 0.0068; P for nonlinear = 0.2433) and cardiovascular disease mortality (P for overall = 0.13; P for nonlinear = 0.4157), with no evidence of a non-linear relationship (Fig. 3).

**Fig. 4:**
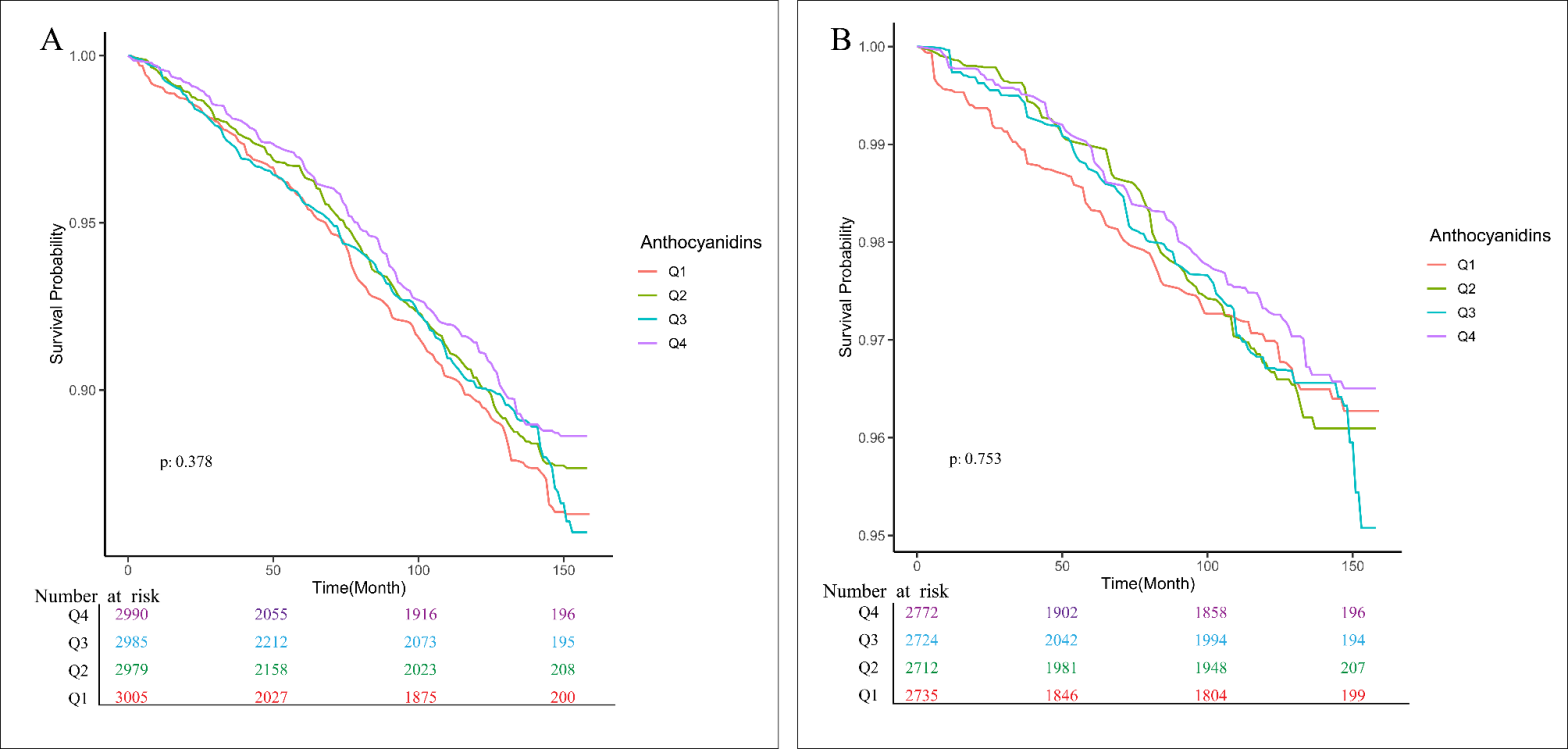
Kaplan-Meier Survival Curves in relationship with anthocyanidins intake. **A** For all-cause mortality, **B** for cardiovascular diseases mortality.

**Fig. 5:**
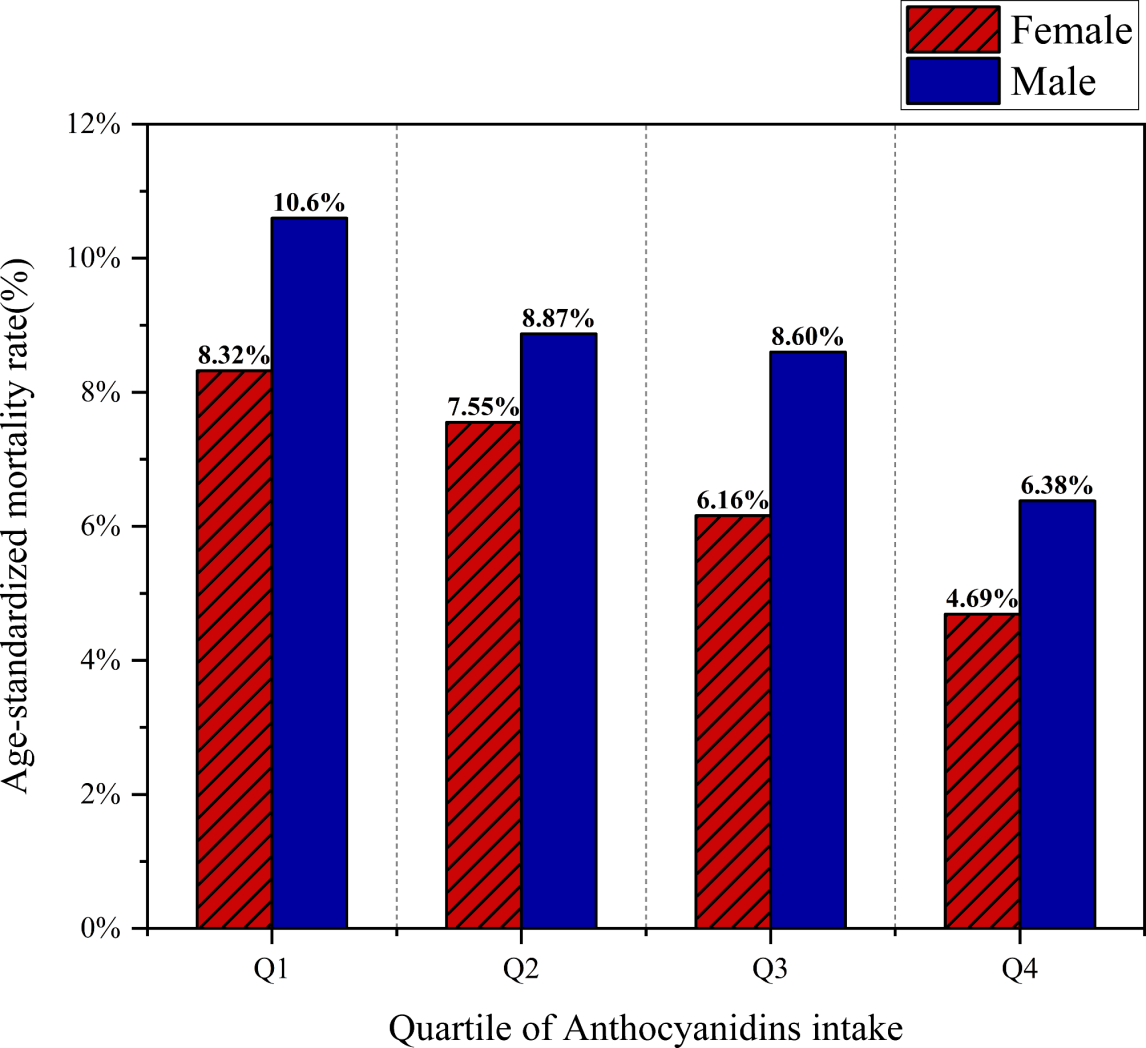
The relationship stratified by gender between age-standardized mortality rates and anthocyanin intake.

The Kaplan-Meier survival curve assessment revealed that participants in the top quartile of anthocyanin consumption (Q4) demonstrated reduced rates of overall mortality and cardiovascular mortality when contrasted with those in the remaining three quartiles (Fig. 4). Age-standardized mortality rates, calculated based on the 2000 census, revealed that men and women in the lowest quartile of anthocyanin intake (Q1) had age-standardized mortality rates of 10.6% and 8.32%, respectively. In contrast, those in the higher level of anthocyanin intake (Q4) demonstrated substantially lower age-standardized mortality rates, at 6.38% for men and 4.69% for women (Fig. 5).

### Subgroup and sensitivity analyses

**Fig. 6:**
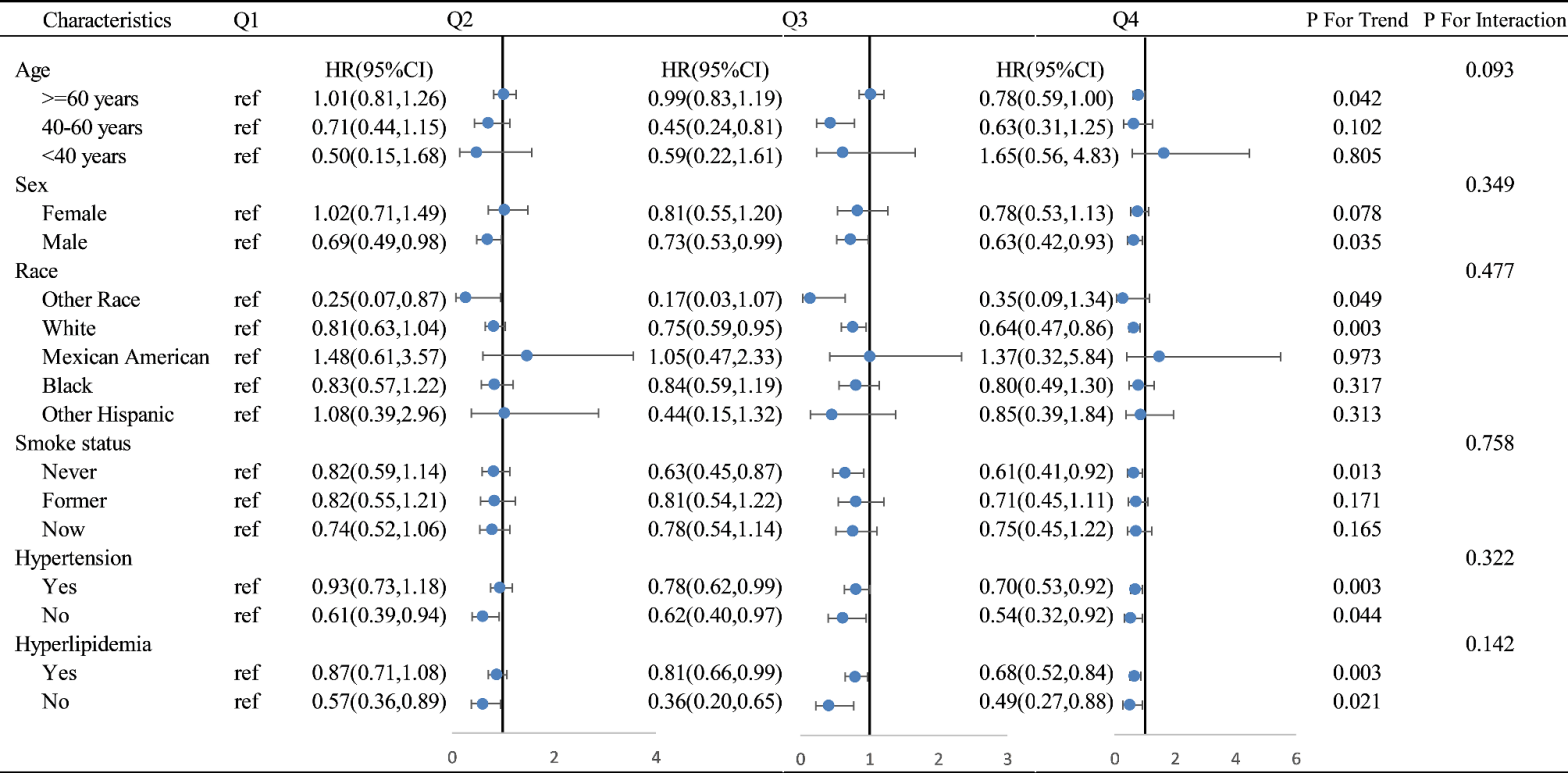
Associations between anthocyanin intake and all-cause mortality stratified by baseline age, sex, race, smoking status, hypertension and hyperlipidemia. *Covariate adjustment using a fully adjusted model: age, alcohol consumption, sex, ethnicity, smoke, body mass index, education, healthy eating index score, Hypertension, Diabetes mellitus, Hyperlipidemia, total energy, protein, carbohydrate and sugars intake.

The stratified assessment examining the relationship between dietary anthocyanin intake and all-cause mortality revealed consistent findings across various subgroups. These subgroups were stratified by age (≥ 60, 40–60, < 40 years), sex, race, smoking status, hypertension, and hyperlipidemia after multivariable adjustments. There were no significant interactions observed among these subgroups (*P* > 0.05). However, significant linear trends were detected within specific subgroups: individuals aged ≥ 60 years (*p* = 0.042), males (*p* = 0.035), whites (*p* = 0.003), never-smokers (*p* = 0.013), those with hypertension (*p* = 0.003), and those with hyperlipidemia (*p* = 0.003) during the trend tests. (Fig. 6)

In the sensitivity analysis, omitting individuals who had a follow-up duration shorter than 24 months did not alter the consistent results regarding anthocyanins and all-cause mortality. Nonetheless, there were minor variations in the results for cardiovascular mortality within the multiple-adjusted models. (Supplemental Table 2)

## Discussion

Within the framework of this prospective cohort investigation, part of the NHANES involving United States adults, we observed that elevated dietary anthocyanidin intake tied to a diminished risk of all-cause mortality, exhibiting dose-dependent protective effects. The age-adjusted hazard ratio (HR) and multivariate adjustment HR for all-cause mortality in the higher level of anthocyanidin consumption were 0.48 (95% CI: 0.39–0.59) and 0.68 (95% CI: 0.52–0.89), respectively. For cardiovascular disease mortality, the age-adjusted HR and multivariate adjustment HR were 0.44 (95% CI: 0.30–0.65) and 0.61 (95% CI: 0.38–0.97) in the respective sequence. Moreover, analyzing the trend relationship between increasing quartiles of anthocyanin intake and both all-cause and cardiovascular disease-specific mortality revealed a significant linear trend (*P* < 0.05). Therefore, we employed RCS to explore the dose–response relationship, which identified a consistent decreasing trend in both all-cause and cardiovascular disease mortality with higher anthocyanidin intake, with devoid of nonlinear associations.

In the stratified analyses, a significant association between the highest quartile dietary anthocyanidin intake in the quartile group and all-cause mortality was found in males [HR = 0.63, 95%CI: (0.42–0.93)] but without statistical significance in females. Simultaneously, notable associations were found in subgroups of never smokers [HR = 0.61, 95% CI: (0.41–0.92)] and individuals of white ethnicity [HR = 0.64, 95% CI: (0.47–0.86)], remaining significant regardless of the presence of hypertension or hyperlipidemia (*P* < 0.05). In addition, we used dietary anthocyanidin intakes to demonstrate the intake levels and trends of dietary anthocyanin different demographic groups with distinct characteristics (Fig. 2). In US adults, the total intake of anthocyanidin gradually increases with a rise in educational background, namely from Less Than 9th Grade to College Graduate or above. In the same way, the trend of anthocyanidin intake shows an increasing fluctuation with age.

Kaplan-Meier survival curve analysis demonstrated that participants in the uppermost quartile of anthocyanin consumption (Q4) showed reduced overall mortality and cardiovascular mortality rates when contrasted with those in the bottom quartile of anthocyanidin intake (Q1). In addition, the age-standardized mortality rate in accordance with the 2000 census decreased with a higher intake of anthocyanidin; the age-standardized mortality rate was 4.69% among those with the highest intake of anthocyanidin in female. There was a 3.63% reduction compared to the lowest quartile of anthocyanidin intake in female (Fig. 5).

Previous epidemiological research has explored the correlation between anthocyanin-rich foods (such as grapes, blueberries, blackberries, and other pigmented berries) and human health. Mink et al.^34^ conducted a prospective study that spanned 16 years, focusing on postmenopausal women. Their findings revealed that participants who consumed strawberries or blueberries at least once a week experienced a significantly lower risk of cardiovascular disease-related mortality. Similarly, in a cohort study conducted in France^35^, the cardioprotective benefits of wine were noted. This study was the first to identify the “French Paradox,” which suggests that moderate red wine consumption is linked to a reduced risk of coronary heart disease. In a study conducted on American adults^12^, it was observed that that increasing daily anthocyanin intake to no more than 20 milligrams might strengthen their protective effects against lung cancer. But the dose–response analysis suggested that the effective anthocyanin intake for preventing lung cancer is unlikely to exceed 20 milligrams per day. However, there is currently no prospective study on the total intake of anthocyanins and all-cause mortality or cardiovascular mortality. This lack of research has prevented the acquisition of convincing epidemiological evidence on dietary anthocyanin intake levels. Therefore, we initiated a study to address this gap and to explore possible mechanisms overlooked in prior research.

In addition to the epidemiological evidence, various experimental studies at different levels further elucidate the mechanisms by which anthocyanin intake confers protective benefits across multiple human body systems. Notably, anthocyanins demonstrate antioxidant capabilities by actively neutralizing reactive oxygen species (ROS), within biological systems^36^. Furthermore, anthocyanins indirectly contribute to anti-aging processes by enhancing the activities of superoxide dismutase (SOD) and catalase (CAT)^37^. A French in vitro study^38^ found that anthocyanins have anti-apoptotic effects in aortic endothelial cells through the NO-guanylate cyclase pathway. Simultaneously, anthocyanins upregulate endothelial nitric oxide synthase (eNOS) expression via the MEK1/2 pathway, significantly increasing nitric oxide (NO) production by the eNOS isoform. This upregulation plays a pivotal role in safeguarding cardiovascular endothelial cells from apoptosis. Additionally, several studies indicate that anthocyanins can activate nuclear factor erythroid 2-related factor 2 (Nrf2). Through the heme oxygenase-1 (HO-1) pathway^39,40^, anthocyanins induce extensive Nrf2 expression in high oxygen-consuming organs such as the heart, lungs, and brain^41,42^. In the response to oxidative stress and inflammation, Nrf2 accumulates in the cell nucleus and triggers protective defenses, thereby bolstering cellular resistance to oxidative damage.

To date, this study remains the most extensive population-based investigation into the relationship between dietary anthocyanidin intake with all-causes mortality and cardiovascular diseases mortality in adults. Nevertheless, some limitations should be considered in the interpretation of our findings. Firstly, in comparison to interventional studies, cohort studies based on observational data face significant challenges in completely eliminating potential confounding factors, thereby limiting their ability to establish causality. Additionally, considering that red wine is a notable source of anthocyanins, there is insufficient evidence regarding the potential synergistic effects of simultaneous alcohol and anthocyanin consumption. Furthermore, dietary anthocyanin intake was obtained through 24-hour dietary recalls; the potential recall bias was challenging to overcome. Moreover, our findings are confined to the analysis of anthocyanin intake and do not include the evaluation of anthocyanin plasma levels. To better mirror real-world scenarios, this study exclusively focuses on the intake of anthocyanins from fresh food sources, deliberately excluding any intake from anthocyanin supplements.

## Conclusions

In summary, we found that an inverse relationship between anthocyanin intake and the risk of all-cause mortality among U.S. adults. We observed a similar inverse relationship between cardiovascular disease (CVD) mortality and anthocyanin intake, suggesting a potential reduction in CVD-related deaths. In the RCS regression analysis, the dose–response curve demonstrated a monotonic decrease in both all-cause mortality and CVD mortality with increasing anthocyanin consumption. These results imply that dietary anthocyanin intake may confer protective effects against all-cause and CVD mortality, thereby recommending the incorporation of anthocyanin-rich foods into the diets of specific populations.

## Electronic supplementary material

Below is the link to the electronic supplementary material.

Supplementary Material 1

Supplementary Material 2

Supplementary Material 3

## Data Availability

Publicly available datasets were analyzed in this study. These data can be found here: https://www.cdc.gov/nchs/nhanes/index.htm. And the datasets have analysed during the current study are available from the corresponding author on reasonable request.

## References

[1] Nyman, N. A. & Kumpulainen, J. T. Determination of anthocyanidins in berries and red wine by high-performance liquid chromatography. *J. Agric. Food Chem.*** 49**(9), 4183–4187 (2001). 11559107 10.1021/jf010572i

[2] Truong, V. D. et al. Characterization of anthocyanins and anthocyanidins in purple-fleshed sweetpotatoes by HPLC-DAD/ESI-MS/MS. *J. Agric. Food Chem.*** 58**(1), 404–410 (2010). 20017481 10.1021/jf902799a

[3] Wang, B. et al. Anti-aging effects and mechanisms of anthocyanins and their intestinal microflora metabolites. *Crit. Rev. Food Sci. Nutr.*** 64**(8), 2358–2374 (2024). 36128763 10.1080/10408398.2022.2123444

[4] Yousuf, B., Gul, K., Wani, A. A. & Singh, P. Health benefits of anthocyanins and their encapsulation for potential use in Food systems: a review. *Crit. Rev. Food Sci. Nutr.*** 56**(13), 2223–2230 (2016). 25745811 10.1080/10408398.2013.805316

[5] Bars-Cortina, D., Sakhawat, A., Piñol-Felis, C. & Motilva, M. J. Chemopreventive effects of anthocyanins on colorectal and breast cancer: a review. *Semin. Cancer Biol.*** 81**, 241–258 (2022). 33359264 10.1016/j.semcancer.2020.12.013

[6] Posadino, A. M. et al. An updated overview of cyanidins for chemoprevention and cancer therapy. *Biomed. Pharmacother.* **163**, 114783 (2023). 10.1016/j.biopha.2023.114783 37121149

[7] Panchal, S. K. & Brown, L. Potential benefits of anthocyanins in chronic disorders of the central nervous system. *Molecules (Basel Switzerland)*** 28**(1), 80 (2022). 10.3390/molecules28010080 PMC9822395 36615279

[8] Ullah, R., Khan, M., Shah, S. A., Saeed, K. & Kim, M. O. Natural antioxidant anthocyanins—a hidden therapeutic candidate in metabolic disorders with major focus in neurodegeneration. *Nutrients*** 11**(6), 1195 (2019). 10.3390/nu11061195 PMC6628002 31141884

[9] Sogari, G., Velez-Argumedo, C., Gómez, M. I. & Mora, C. College students and eating habits: a study using an ecological model for healthy behavior. *Nutrients*** 10**(12), 1823 (2018). 10.3390/nu10121823 PMC6315356 30477101

[10] Srour, B. et al. Ultra-processed food intake and risk of cardiovascular disease: prospective cohort study (NutriNet-Santé). BMJ (Clin. Res. ed.)** 365**, 1451 (2019). 10.1136/bmj.l1451 PMC6538975 31142457

[11] Lane, M. M. et al. Ultra-processed food exposure and adverse health outcomes: umbrella review of epidemiological meta-analyses. *BMJ (Clin. Res. ed.)*** 384**, e077310 (2024). 10.1136/bmj-2023-077310 PMC10899807 38418082

[12] Zhang, Y., Zhu, M., Wan, H., Chen, L. & Luo, F. Association between dietary anthocyanidins and risk of lung cancer. *Nutrients*** 14**(13), 2643 (2022). 10.3390/nu14132643 PMC9268346 35807824

[13] Xu, X., Zhu, Y., Li, S. & Xia, D. Dietary intake of anthocyanidins and renal cancer risk: a prospective study. *Cancers*** 15**(5), 1406 (2023). 10.3390/cancers15051406 PMC10001018 36900199

[14] Adu, M. D. et al. Association between non-tea flavonoid intake and risk of type 2 diabetes: the Australian diabetes, obesity and lifestyle study. *Food Funct.*** 13**(8), 4459–4468 (2022). 35380573 10.1039/d1fo04209b

[15] Yang, L. et al. Role of purified anthocyanins in improving cardiometabolic risk factors in Chinese men and women with prediabetes or early untreated diabetes—a randomized controlled trial. *Nutrients*** 9**(10), 1104 (2017). 10.3390/nu9101104 PMC5691720 28994705

[16] Cassidy, A. et al. Habitual intake of anthocyanins and flavanones and risk of cardiovascular disease in men. *Am. J. Clin. Nutr.*** 104**(3), 587–594 (2016). 27488237 10.3945/ajcn.116.133132 PMC4997299

[17] Curtis, P. J. et al. Blueberries improve biomarkers of cardiometabolic function in participants with metabolic syndrome-results from a 6-month, double-blind, randomized controlled trial. *Am. J. Clin. Nutr.*** 109**(6), 1535–1545 (2019). 31136659 10.1093/ajcn/nqy380 PMC6537945

[18] Tran, P. H. L. & Tran, T. T. D. Blueberry supplementation in neuronal health and protective technologies for efficient delivery of blueberry anthocyanins. *Biomolecules*** 11**(1), 102 (2021). 10.3390/biom11010102 PMC7828789 33466731

[19] Cheng, J. R., Liu, X. M., Chen, Z. Y., Zhang, Y. S. & Zhang, Y. H. Mulberry anthocyanin biotransformation by intestinal probiotics. *Food Chem.*** 213**, 721–727 (2016). 27451240 10.1016/j.foodchem.2016.07.032

[20] Traustadóttir, T. et al. Tart cherry juice decreases oxidative stress in healthy older men and women. *J. Nutr.*** 139**(10), 1896–1900 (2009). 19692530 10.3945/jn.109.111716 PMC3151016

[21] Jacques, P. F., Cassidy, A., Rogers, G., Peterson, J. J. & Dwyer, J. T. Dietary flavonoid intakes and CVD incidence in the Framingham offspring cohort. *Br. J. Nutr.*** 114**(9), 1496–1503 (2015). 26334117 10.1017/S0007114515003141 PMC4613998

[22] Lai, H. T. et al. Fruit intake and cardiovascular disease mortality in the UK women’s Cohort Study. *Eur. J. Epidemiol.*** 30**(9), 1035–1048 (2015). 26076918 10.1007/s10654-015-0050-5

[23] Chen, F. et al. Association among dietary supplement use, nutrient intake, and mortality among U.S. adults: a cohort study. *Ann. Intern. Med.*** 170**(9), 604–613 (2019). 30959527 10.7326/M18-2478 PMC6736694

[24] Menconi, J., Perata, P. & Gonzali, S. In pursuit of purple: anthocyanin biosynthesis in fruits of the tomato clade. *Trends Plant Sci.*** 29**(5), 589–604 (2024). 38177013 10.1016/j.tplants.2023.12.010

[25] Liese, A. D. et al. The dietary patterns methods project: synthesis of findings across cohorts and relevance to dietary guidance. *J. Nutr.*** 145**(3), 393–402 (2015). 25733454 10.3945/jn.114.205336 PMC4336525

[26] Krebs-Smith, S. M. et al. Update of the healthy eating index: HEI-2015. *J. Acad. Nutr. Diet.*** 118**(9), 1591–1602 (2018). 30146071 10.1016/j.jand.2018.05.021 PMC6719291

[27] Third Report of the National Cholesterol Education Program (NCEP). Expert panel on detection, evaluation, and treatment of high blood cholesterol in adults (adult treatment panel III) final report. *Circulation*** 106**(25), 3143–3421 (2002). 12485966

[28] Lau, E., Neves, J. S., Ferreira-Magalhães, M., Carvalho, D. & Freitas, P. Probiotic ingestion, obesity, and metabolic-related disorders: results from NHANES, 1999–2014. *Nutrients*** 11**(7), 1482 (2019). 10.3390/nu11071482 PMC6683043 31261830

[29] Rattan, P. et al. Inverse association of telomere length with liver disease and mortality in the US population. *Hepatol. Commun.*** 6**(2), 399–410 (2022). 34558851 10.1002/hep4.1803 PMC8793996

[30] Hicks, C. W., Wang, D., Matsushita, K., Windham, B. G. & Selvin, E. Peripheral neuropathy and all-cause and cardiovascular mortality in U.S. adults: a prospective cohort study. *Ann. Intern. Med.*** 174**(2), 167–174 (2021). 33284680 10.7326/M20-1340 PMC7932559

[31] Mancia, G. et al. 2023 ESH guidelines for the management of arterial hypertension the task force for the management of arterial hypertension of the European Society of Hypertension: endorsed by the International Society of Hypertension (ISH) and the European Renal Association (ERA). *J. Hypertens.*** 41**(12), 1874–2071 (2023). 37345492 10.1097/HJH.0000000000003480

[32] World Health Organization & International Diabetes Federation. (2006). Definition and diagnosis of diabetes mellitus and intermediate hyperglycaemia: report of a WHO/IDF consultation.World Health Organization. https://iris.who.int/handle/10665/43588

[33] ElSayed, N. A. et al. Introduction and methodology: standards of care in diabetes-2023. *Diabetes care*** 46**(Suppl 1), S1–S4 (2023). 36507647 10.2337/dc23-Sint PMC9810461

[34] Mink, P. J. et al. Flavonoid intake and cardiovascular disease mortality: a prospective study in postmenopausal women. *Am. J. Clin. Nutr.*** 85**(3), 895–909 (2007). 17344514 10.1093/ajcn/85.3.895

[35] Renaud, S. & de Lorgeril, M. Wine, alcohol, platelets, and the French paradox for coronary heart disease. *Lancet (Lond. Engl.)*** 339**(8808), 1523–1526 (1992). 10.1016/0140-6736(92)91277-f 1351198

[36] Matera, R. et al. Acylated anthocyanins from sprouts of Raphanus sativus Cv. Sango: isolation, structure elucidation and antioxidant activity. *Food Chem.*** 166**, 397–406 (2015). 25053073 10.1016/j.foodchem.2014.06.056

[37] Lu, X., Zhou, Y., Wu, T. & Hao, L. Ameliorative effect of black rice anthocyanin on senescent mice induced by D-galactose. *Food Funct.*** 5**(11), 2892–2897 (2014). 25190075 10.1039/c4fo00391h

[38] Martin, S., Giannone, G., Andriantsitohaina, R. & Martinez, M. C. Delphinidin, an active compound of red wine, inhibits endothelial cell apoptosis via nitric oxide pathway and regulation of calcium homeostasis. *Br. J. Pharmacol.*** 139**(6), 1095–1102 (2003). 12871827 10.1038/sj.bjp.0705347 PMC1573941

[39] Sorrenti, V. et al. Heme oxygenase induction by cyanidin-3-o-beta-glucoside in cultured human endothelial cells. *Mol. Nutr. Food Res.*** 51**(5), 580–586 (2007). 17440991 10.1002/mnfr.200600204

[40] Parzonko, A., Oświt, A., Bazylko, A. & Naruszewicz, M. Anthocyans-rich Aronia melanocarpa extract possesses ability to protect endothelial progenitor cells against angiotensin II induced dysfunction. *Phytomed. Int. J. Phytother. Phytopharmacol.*** 22**(14), 1238–1246 (2015). 10.1016/j.phymed.2015.10.009 26655406

[41] Aboonabi, A. & Singh, I. Chemopreventive role of anthocyanins in atherosclerosis via activation of Nrf2-ARE as an indicator and modulator of redox. *Biomed. Pharmacother*. **72**, 30–36 (2015). 26054672 10.1016/j.biopha.2015.03.008

[42] Chen, B., Lu, Y., Chen, Y. & Cheng, J. The role of Nrf2 in oxidative stress-induced endothelial injuries. *J. Endocrinol.*** 225**(3), R83–99 (2015). 25918130 10.1530/JOE-14-0662

