# Supplementary material for: Association of dietary anthocyanidins intake with all-cause mortality and cardiovascular diseases mortality in USA adults: a prospective cohort study
Source: Sci Rep. 2024 Nov 4;14:26595. doi: 10.1038/s41598-024-76805-z (PMC11535342; doi:10.1038/s41598-024-76805-z)
Supplement: Supplementary file 1 — Supplementary Material 1 [file 41598_2024_76805_MOESM1_ESM.docx]

1. **Supplementary methods**

Hyperlipidemia was diagnosed by:

- Hypertriglyceridemia: TG ≥ 150mg/dL.
- Hypercholesterolemia:
- total cholesterol (TC) ≥ 200 mg/dL[5.18mmol/L]
- low-density lipoprotein (LDL) ≥ 130 mg/dL[3.37mmol/L]
- high-density lipoprotein (HDL) ≤ 40 mg/dL[1.04mmol/L] in males, ≤ 50 mg/dL[1.30mmol/L] in females
- Use of lipid-lowering drugs.

Diabetes mellitus was diagnosed by:

- Self-reported doctor diagnosis of diabetes.
- Glycohemoglobin HbA1c > 6.5%
- Fasting glucose ≥7.0 mmol/L.
- Random blood glucose ≥11.1 mmol/L.
- Two-hour OGTT blood glucose ≥ 11.1 mmol/L.
- Use of diabetes medication or insulin.

Impaired Fasting Glycaemia (IFG) was diagnosed by:

- 6.1 mmol/L ≤ Fasting glucose ≤ 7.0 mmol/L.

Impaired Glucose Tolerance (IGT) was diagnosed by:

- 7.8 mmol/l ≤ Two-hour OGTT blood glucose ≤ 11.1 mmol/l.

Hypertension was diagnosed by

- Blood pressure measurements taken three or more times on different days all show that: systolic blood pressure (SBP) ≥ 140 mmHg or diastolic blood pressure (DBP) ≥ 90 mmHg
- self-reported diagnosis
- use of antihypertensive drugs

Alcohol consumption status was defined as:

- never (had <12 drinks in lifetime)
- former (had ≥12 drinks in 1 year and did not drink last year, or did not drink last year but drank ≥12 drinks in lifetime)
- mild (≥1 drinks per day for females, ≥2 drinks per day for males)
- moderate (≥2 drinks per day for females, ≥3 drinks per day for males, or binge drinking [≥4 drinks on same occasion for females, ≥5 drinks on same occasion for males] ≥2 days per month)
- heavy (≥3 drinks per day for females, ≥4 drinks per day for males, or binge drinking on 5 or more days per month)

Smoking status was defined as:

- now (smoked moth than 100 cigarettes in life and smoke some days or every day)
- former (smoked more than 100 cigarettes in life and smoke not at all now)
- never (smoked less than 100 cigarettes in life)
