# Supplementary material for: Association of dietary anthocyanidins intake with all-cause mortality and cardiovascular diseases mortality in USA adults: a prospective cohort study
Source: Sci Rep. 2024 Nov 4;14:26595. doi: 10.1038/s41598-024-76805-z (PMC11535342; doi:10.1038/s41598-024-76805-z)
Supplement: Supplementary file 2 — Supplementary Material 2 [file 41598_2024_76805_MOESM2_ESM.docx]

| **Supplementary Table 1** Characteristics of study participants according to survival condition | | | |
| --- | --- | --- | --- |
| **variable^a^** | **alive**^b^ | **death**^b^ | **Pvalue** |
| **body mass index** | 29.12±0.14 | 29.58±0.33 | 0.24 |
| **Healthy eating index score** | 50.70±0.39 | 51.79±0.42 | 0.02 |
| **Age (years)** | 45.45±0.34 | 66.96±0.52 | < 0.0001 |
| **Total energy (kcal/day)** | 2122.81±14.35 | 1818.26±32.25 | < 0.0001 |
| **Protein (g/day)** | 83.30±0.62 | 70.79±1.30 | < 0.0001 |
| **Carbohydrate (g/day** | 251.87±1.76 | 224.21±4.08 | < 0.001 |
| **Total sugars (g/day)** | 111.03±1.13 | 101.64±2.66 | < 0.001 |
| **Total Anthocyanidins (mg/day)** | 14.68±0.86 | 11.23±1.13 | 0.01 |
| **Alcohol user** |  |  | < 0.0001 |
| Former | 1274(9.40) | 501(32.64) |  |
| Heavy | 2277(22.85) | 139(10.71) |  |
| Mild | 3818(39.11) | 444(33.54) |  |
| Moderate | 1809(18.84) | 107(8.57) |  |
| Never | 1360(9.80) | 230(14.55) |  |
| **Sex** |  |  | 0.04 |
| Female | 5541(52.16) | 604(47.77) |  |
| Male | 4997(47.84) | 817(52.23) |  |
| **Ethnicity** |  |  | < 0.0001 |
| Mexican American | 1780(8.68) | 102(3.36) |  |
| Black | 2140(11.20) | 275(11.35) |  |
| White | 4646(67.29) | 916(79.73) |  |
| Other Hispanic | 1118(5.78) | 92(2.33) |  |
| Other Race | 854(7.06) | 36(3.23) |  |
| **Education** |  |  | < 0.0001 |
| 9-11th grade | 1433(9.54) | 274(18.43) |  |
| College Graduate or above | 2462(30.64) | 212(18.32) |  |
| High school graduate/GED | 2457(24.64) | 387(26.72) |  |
| Less Than 9th Grade | 980(4.16) | 229(12.04) |  |
| Some college or AA degree | 3206(31.02) | 319(24.49) |  |
| **smoke** |  |  | < 0.0001 |
| Former | 2433(23.34) | 573(38.27) |  |
| Never | 5969(57.72) | 542(38.49) |  |
| Now | 2136(18.94) | 306(23.24) |  |
| **Physical activity level** |  |  | < 0.0001 |
| Very low | 2500(18.93) | 669(42.37) |  |
| Low | 2693(25.37) | 345(25.50) |  |
| Intermediate | 2585(27.66) | 237(18.79) |  |
| High | 2760(28.03) | 170(13.34) |  |
| **Hypertension** |  |  | < 0.0001 |
| No | 6521(67.25) | 432(33.69) |  |
| Yes | 4017(32.75) | 989(66.31) |  |
| **DM** |  |  | < 0.0001 |
| DM^c^ | 1723(11.99) | 529(33.07) |  |
| IFG^c^ | 528(4.60) | 77(5.59) |  |
| IGT^c^ | 332(2.62) | 71(5.57) |  |
| no | 7955(80.79) | 744(55.76) |  |
| **Hyperlipidemia** |  |  | < 0.0001 |
| No | 3117(31.75) | 277(18.75) |  |
| Yes | 7421(68.25) | 1144(81.25) |  |

^a^Values are numbers (%) for categorical variables and means ± SDs for continuous variables

^b^The NHANES participants was weighted differently to adjust for the probability of cluster sampling and oversampling of Hispanics and African Americans or aged 60 and above. Therefore the actual number does not match the weighted percentage

^c^ DM, Diabetes mellitus; IFG, Impaired Fasting Glycaemia; IGT, Impaired Glucose Tolerance
