# Supplementary material for: Association of dietary anthocyanidins intake with all-cause mortality and cardiovascular diseases mortality in USA adults: a prospective cohort study
Source: Sci Rep. 2024 Nov 4;14:26595. doi: 10.1038/s41598-024-76805-z (PMC11535342; doi:10.1038/s41598-024-76805-z)
Supplement: Supplementary file 3 — Supplementary Material 3 [file 41598_2024_76805_MOESM3_ESM.docx]

**Supplementary Table 2** Weighted association between Isoflavones and mortality when excluding participants with less than 24 months of follow-up

| Characteristic | Quintiles of dietary anthocyanidins intake | | | | P-Trend |
| --- | --- | --- | --- | --- | --- |
|  | Q1 | Q2 | Q3 | Q4 |  |
| Participants, No. | 2558 | 2558 | 2555 | 2556 | NA |
| Followtime, years | 9.11±0.32 | 9.20±0.28 | 9.05±0.32 | 8.57±0.36 |  |
| Anthocyanidins intake, median(Range), mg/d | 0.00  [0.00,0.16] | 0.995  (0.16,2.19] | 4.885  (2.19,11.42] | 27.42 (11.42,756.10] | NA |
| All-cause mortality |  |  |  |  |  |
| Death/person-years | 325/2,181 | 307/2,071 | 303/2,088 | 277/1,915 |  |
| Model1[HR (95 % CI)] ^a^ | Referent | 0.75(0.59,0.96) | 0.60(0.46,0.78) | 0.50(0.40,0.63) | <0.0001 |
| Model2[HR (95 % CI)] ^b^ | Referent | 0.84(0.67,1.06) | 0.74(0.57,0.98) | 0.68(0.52,0.89) | 0.003 |
| Model3[HR (95 % CI)] ^c^ | Referent | 0.84(0.66,1.05) | 0.74(0.56,0.96) | 0.71(0.53,0.94) | 0.011 |
| Cardiovascular mortality |  |  |  |  |  |
| Death/person-years | 87/579 | 85/556 | 92/618 | 89/613 |  |
| Model1[HR (95 % CI)] ^a^ | Referent | 0.93(0.58,1.51) | 0.68(0.46,0.99) | 0.51(0.36,0.74) | <0.0001 |
| Model2[HR (95 % CI)] ^b^ | Referent | 1.07(0.67, 1.71) | 0.94(0.66, 1.34) | 0.79(0.56, 1.13) | 0.106 |
| Model3[HR (95 % CI)] ^c^ | Referent | 1.00(0.63, 1.59) | 0.87(0.61, 1.23)) | 0.72(0.48, 1.09) | 0.057 |

^a^Cox proportional hazard model adjusted for age

^b^Cox proportional hazards model adjusted by age sex, ethnicity, smoke, BMI, education level, physical activity level , total energy intake, protein intake, carbohydrate intake, sugars intake

^c^Further adjusted for alcohol user, healthy eating index, Hypertension, Diabetes mellitus, Hyperlipidemia
